# Non-contrast MRI can accurately characterize adnexal masses: a retrospective study

**DOI:** 10.1007/s00330-021-07737-9

**Published:** 2021-03-16

**Authors:** Hilal Sahin, Camilla Panico, Stephan Ursprung, Vittorio Simeon, Paolo Chiodini, Amy Frary, Bruno Carmo, Janette Smith, Sue Freeman, Mercedes Jimenez-Linan, Helen Bolton, Krishnayan Haldar, Joo Ern Ang, Caroline Reinhold, Evis Sala, Helen Addley

**Affiliations:** 1 Department of Radiology, School of Clinical Medicine, University of Cambridge, Cambridgeshire, Cambridge, CB2 0QQ UK; 2 Cancer Research UK Cambridge Centre, University of Cambridge, Cambridge, UK; 3 General Diagnostic and Interventional Radiology, Diagnostic Imaging Area, Department of Diagnostic Imaging, Radiation Oncology and Hematology, Fondazione Policlinico Universitario “A. Gemelli”-IRCCS, Universita Cattolicá del Sacro Cuore, Rome, Italy; 4 Medical Statistics Unit, Department of Mental, Physical Health and Preventive Medicine, University of Campania Luigi Vanvitelli, Naples, Italy; 5 Department of Radiology, Addenbrooke’s Hospital and University of Cambridge, Cambridge, UK; 6 Department of Histopathology, Addenbrooke’s Hospital, Cambridge, UK; 7 Gynaecological Oncology, Addenbrooke’s Hospital, Cambridge, UK; 8 SGRN, Surgical Gynaecological Oncology Research Network, UK; 9 Department of Oncology, Addenbrooke’s Hospital, Cambridge, UK; 10 Department of Medical Imaging, McGill University Health Centre (MUHC), Montreal, Quebec Canada; 11 Augmented Intelligence Precision Laboratory (AIPHL), McGill University Health Centre Research Institute, Montreal, Quebec Canada

**Keywords:** Diagnosis, Magnetic resonance imaging, Ovarian cancer, Sensitivity and specificity

## Abstract

**Objective:**

To determine the accuracy of interpretation of a non-contrast MRI protocol in characterizing adnexal masses.

**Methods and materials:**

Two hundred ninety-one patients (350 adnexal masses) who underwent gynecological MRI at our institution between the 1^st^ of January 2008 and the 31^st^ of December 2018 were reviewed. A random subset (102 patients with 121 masses) was chosen to evaluate the reproducibility and repeatability of readers’ assessments. Readers evaluated non-contrast MRI scans retrospectively, assigned a 5-point score for the risk of malignancy and gave a specific diagnosis. The reference standard for the diagnosis was histopathology or at least one-year imaging follow-up. Diagnostic accuracy of the *non-contrast MRI score* was calculated. Inter- and intra-reader agreement was analyzed with Cohen’s kappa statistics.

**Results:**

There were 53/350 (15.1%) malignant lesions in the whole cohort and 20/121 (16.5%) malignant lesions in the random subset. Good agreement between readers was found for the *non-contrast MRI score* (*к* = 0.73, 95% confidence interval [CI] 0.58–0.86) whilst the intra-reader agreement was excellent (*к* = 0.81, 95% CI 0.70–0.88). The *non-contrast MRI score* value of ≥ 4 was associated with malignancy with a sensitivity of 84.9%, a specificity of 95.9%, an accuracy of 94.2% and a positive likelihood ratio of 21 (area under the receiver operating curve 0.93, 95% CI 0.90–0.96).

**Conclusion:**

Adnexal mass characterization on MRI without the administration of contrast medium has a high accuracy and excellent inter- and intra-reader agreement. Our results suggest that non-contrast studies may offer a reasonable diagnostic alternative when the administration of intravenous contrast medium is not possible.

**Key Points:**

*• A non-contrast pelvic MRI protocol may allow the characterization of adnexal masses with high accuracy.*

*• The non-contrast MRI score may be used in clinical practice for differentiating benign from malignant adnexal lesions when the lack of intravenous contrast medium precludes analysis with the O*–*RADS MRI score.*

**Supplementary Information:**

The online version contains supplementary material available at 10.1007/s00330-021-07737-9.

## Introduction

The accurate characterization of adnexal masses is critical to guide appropriate patient management. Ultrasound is the primary imaging modality in women with a clinically suspected adnexal mass with 82–92% accuracy [1]. However, approximately 5–20% of adnexal masses remain uncharacterized following US [2, 3]. For these indeterminate masses, although short-term follow-up is an option, MRI is the imaging modality of choice for a rapid characterization [4–6]. Previous papers with differing MRI protocols reported excellent accuracies with a range of 88–93% for the diagnosis of malignancy [7–9].

In 2013, Thomassin-Naggara et al published the ADNEX MR scoring system for risk stratification in adnexal masses [10]. This score proposes a uniform dynamic contrast-enhanced (DCE) MRI protocol and standardized interpretation to be used across centers. Following its publication, the ADNEX scoring system was tested on 1340 women in a prospective multicenter clinical study and integrated into the O–RADS MRI scoring system [11]. The O–RADS MRI score was found to have a sensitivity of 93% and specificity of 91% for stratifying the risk of malignancy in adnexal masses [11]. It is the most comprehensive guidance in the current literature for the characterization of adnexal masses on MRI.

The O–RADS MRI score relies on intravenous gadolinium-based contrast agents (GBCAs) for the assessment of the dynamic enhancement curve [11]. A non-contrast MRI study, therefore, yields an O–RADS MRI score of 0 (incomplete study) [12]. Although the assessment of an adnexal mass using the O–RADS MRI score is recommended by the American College of Radiology [12], there may be situations where avoiding contrast-enhanced MRI is preferable due to logistical and patient factors. Acquisition of the DCE protocol proposed by Thomassin-Naggara et al [10, 11] significantly extends the MRI examination which may be a challenge in certain patients or clinical scenarios. Although Pereira et al recently proposed a simplified dynamic MRI protocol including 5 post-contrast phases with 30-s delays [13, 14], off-line post-processing of dynamic imaging still contributes significantly to the workload. In addition, there are significant concerns regarding the administration of GBCAs in relation to the development of nephrogenic systemic fibrosis and the potential impact of long-term gadolinium retention in a range of tissues and organs [15]. Recently, the Royal College of Radiologists UK published their position statement and recommendations emphasizing that GBCAs should only be used when essential diagnostic information cannot be obtained with unenhanced scans [16, 17].

Therefore, the aim of our study was to evaluate the performance of a non-contrast gynecological MRI protocol in the characterization of adnexal masses and analyze the reproducibility and repeatability.

## Materials and methods

### Patients and study setting

**Fig. 1:**
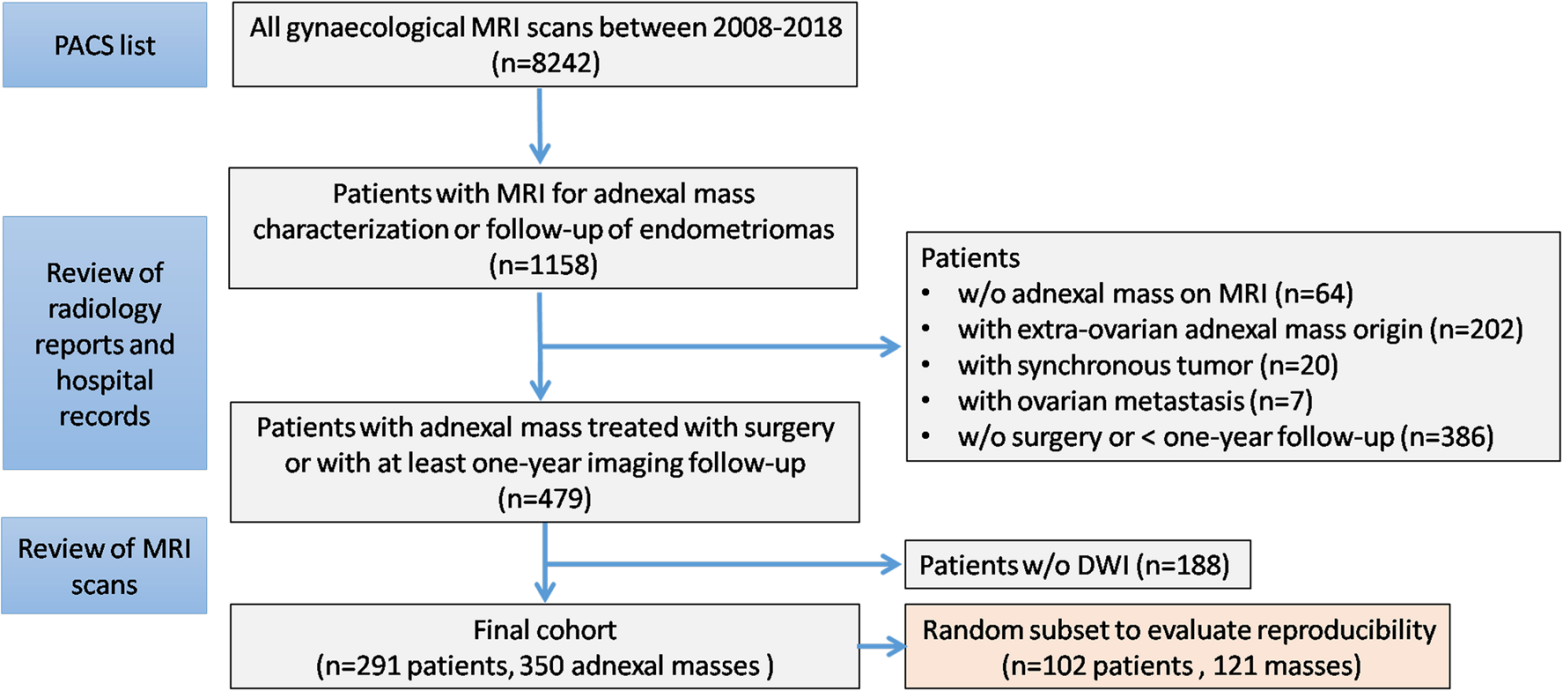
REMARK diagram showing selection of study cohorts. *DWI* diffusion-weighted imaging, *MRI* magnetic resonance imaging, *w/o* without

This single-institution retrospective study was approved by the institutional review board, with the need for informed consent for data analysis waived. Reports of all consecutive gynecological MRI scans from the 1^st^ of January 2008 to the 31^st^ of December 2018 (*n* = 8242) were reviewed. Inclusion criteria were as follows: (1) adult female patients with gynecological MRI performed for adnexal mass characterization or follow-up as recorded on Picture Archiving and Communications System using standard imaging sequences of the female pelvis (Supplementary Table S1), (2) confirmed histopathological diagnosis or at least one-year stability on imaging follow-up. The study flow is summarized in Fig. 1. The final cohort included 291 patients with 350 adnexal masses. From this cohort, 102 patients with 121 adnexal masses stratified for their malignancy status were chosen randomly, to evaluate reproducibility and repeatability of radiologists’ assessments. The patients’ electronic health records were reviewed and CA125 levels were noted, if available. All patients were diagnosed, treated or followed up in the same gynecology oncology department which is a specialist cancer center for gynecological malignancies.

### MRI protocol

MRI examinations were performed on 1.5-T (MR HDx, MR450 Discovery, MR 450 W Optima) and 3-T (MR750 Discovery) MRI systems (all GE Healthcare) using 8-32 channel phased array body coils. Unless contraindicated, hyoscine butylbromide (Buscopan®, Sanofi), 20 mg, was administered i.v. prior to the imaging to reduce peristaltic movement. The MRI protocol for characterization of adnexal masses included sagittal, axial and coronal T_2_-weighted fast spin-echo sequences and axial T_1_-weighted gradient-echo sequences with and without fat-suppression (LAVA-Flex implementation of Dixon method), followed by diffusion-weighted imaging (DWI) with *b* values of 0 and 800–1000 s/mm^2^ (Supplementary Table S1). Apparent diffusion coefficient (ADC) maps were calculated. The MRI protocol used in this study included only non-contrast sequences, post-contrast imaging was not available and complementary use of gadolinium was not assessed.

### Image interpretation and analysis

Two consultant radiologists, with 6 and 8 years of experience in gynecological imaging, took part in image interpretation. Reader 1 assessed the whole cohort (*n* = 291 patients) whilst readers 1 and 2 assessed the random subset (*n* = 102 patients) independently following a 2-month interval to avoid recall bias. The numbers within the cohorts were statistically calculated by power analysis to calculate a sufficient number of patients to assess reproducibility. When multiple masses were present, each lesion was described separately. The readers were blinded to all information except age and CA125 levels.

Morphological features and DWI signal intensity (SI) of the adnexal mass and important accompanying features (ascites, lymphadenopathy or peritoneal implants) were re-evaluated. Morphological MRI features were the presence of a simple cystic mass, purely endometriotic mass, fatty mass, solid mass, multiple septations, thick or irregular septations and solid tissue in the mass. Lymphadenopathy was defined as the enlargement of lymph nodes in the short axis more than 8 mm in the pelvis and 10 mm in the para-aortic region. Solid tissue suspicious for malignancy was defined as tissue within the adnexal mass displaying intermediate SI on T_2_-weighted images, low SI on T_1_-weighted images with corresponding restricted diffusion. True diffusion restriction was defined qualitatively as high SI on high *b* value DWI images and low SI on ADC map. SI of the solid tissue on T_2_- weighted images and ADC map was defined relative to skeletal muscles whilst on DWI, it was compared to cerebrospinal fluid.

**Fig. 2:**
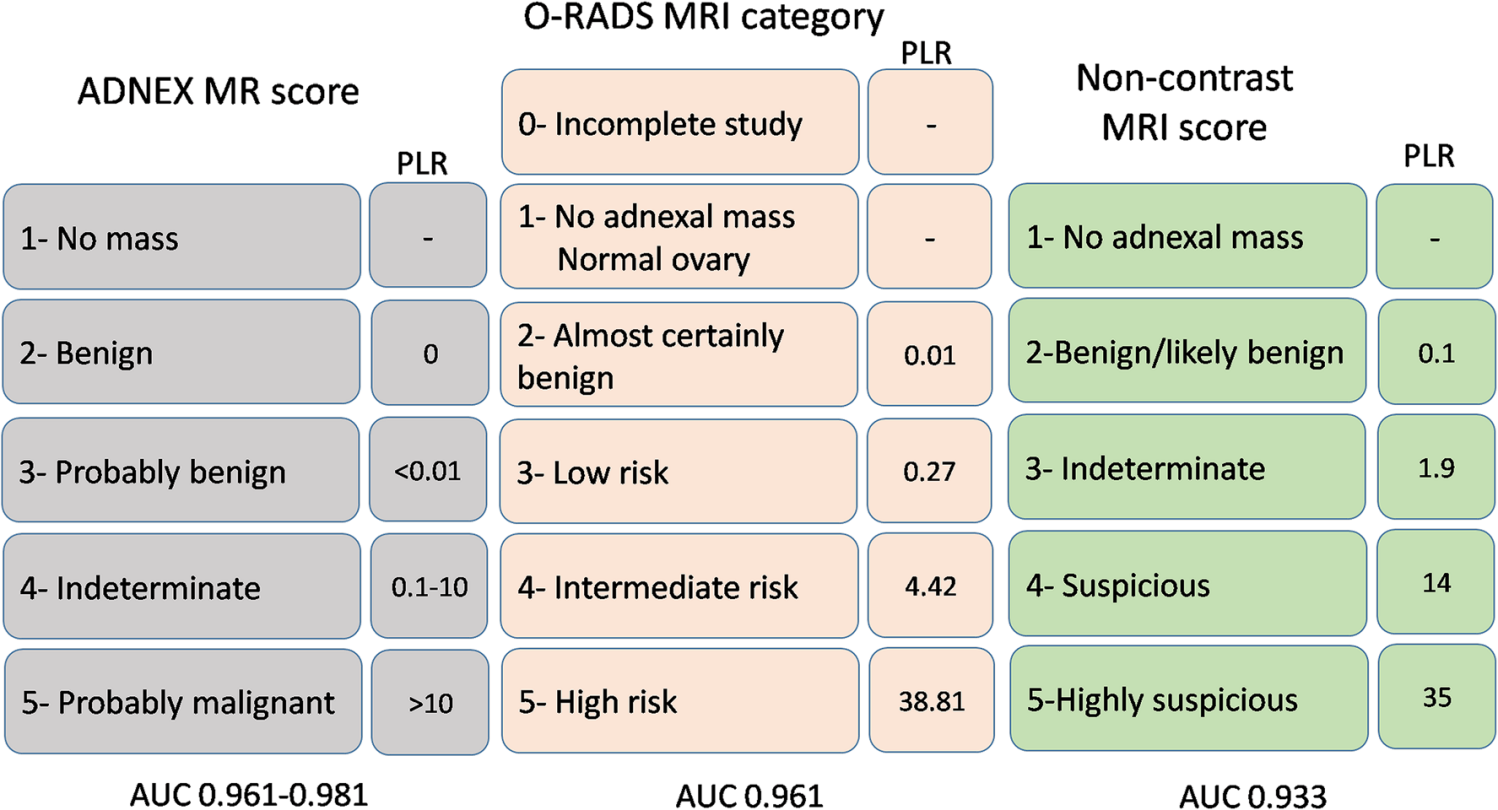
Comparison of ADNEX MR score, O–RADS MRI category and proposed *non-contrast MRI score*. The area under the curve for ADNEX MR score refers to AUCs according to different readers in the training set. *AUC* area under the curve, *MR(I)* magnetic resonance (imaging), *O–RADS* Ovarian–Adnexal Imaging–Reporting and Data System, *PLR* positive likelihood ratio

Readers assigned a score to each adnexal mass using the proposed *non-contrast MRI score* (Fig. 2). The ADNEX MR [10] and O–RADS [11] score informed this 5-point scale. We proposed the following *non-contrast MRI score* (Table 1): 1 = no adnexal mass present; 2 = benign/likely benign and a specific diagnosis was assigned (e.g. endometrioma, dermoid or ovarian fibroma); 3 = indeterminate; 4 = suspicious for malignancy and 5 = highly suspicious for malignancy, i.e. at least one other feature (such as peritoneal implant, ascites or lymphadenopathy) in addition to score 4.

**Table 1:** Non-contrast MRI score

*Non-contrast MRI score*	Definition	MRI features
Score 1	No mass	No adnexal mass is demonstrated in pelvic MRI study
Score 2	Benign/likely benign	Radiologically characterized, has to have a radiological diagnosis (e.g. endometrioma, dermoid, fibroma)
Score 3	Indeterminate	Not classified in other scores. It may have a solid appearing component however this does not reach criteria for solid tissue*
Score 4	Suspicious for malignancy	Solid tissue criteria reached
Score 5	Highly suspicious for malignancy	Solid tissue criteria reached and presence of: • Peritoneal implants and/or • Lymphadenopathy and/or • Ascites in the presence of solid tissue, after benign diagnoses are excluded

*MRI* magnetic resonance imaging

*Solid tissue is defined as tissue with intermediate signal intensity on T2-weighted imaging, low signal intensity on T1-weighted imaging and corresponding true diffusion restriction

In addition to scoring each adnexal mass, readers assigned a specific diagnosis according to their individual evaluation. Agreement between the final and reader assigned diagnosis for the whole cohort and random subset was analyzed.

### Reference standard

Histopathological diagnosis or imaging follow-up for at least 1 year served as the reference standard. Final diagnoses at histopathology were categorized into normal ovary, benign, borderline or malignant disease.

### Statistical analysis

Categorical variables were expressed as absolute numbers and percentages. Continuous variables were described either as median and interquartile range or mean and standard deviation, according to their distribution. *T* test or the Mann-Whitney test for continuous and chi-squared or Fisher’s exact test for categorical variables were used to compare MRI features between benign and malignant tumors. The descriptive analysis on the entire population was based on the assessment of reader 1.

Receiver operating characteristic (ROC) curves, the areas under the curves (AUC) and all conventional measures for diagnostic test accuracy were calculated to assess the *non-contrast MRI score’s* prediction of the reference standard. The final diagnosis was grouped as a binary variable and borderline disease was included in the malignant group. According to a pre-defined cut-off value of score 4, the *non-contrast MRI* score was dichotomized (score ≥ 4 as malignant) to evaluate sensitivity, specificity, accuracy and positive likelihood ratio (PLR). To evaluate inter-reader and intra-reader agreement, Cohen’s kappa (*к*) coefficients and weighted *к* coefficients were computed for ordinal variables. Where appropriate, 95% confidence intervals (CI) were calculated. As described above, a random sample of 102 patients was used to assess the agreement between two raters. Power analysis was performed on the entire patient cohort to calculate a sufficient number of patients to assess reproducibility. This sample size achieves 80% power to detect a true value of 0.75 in a test of H0: *к* = 0.50 vs. H1: *к* < or > 0.50 when there are 4 categories with frequencies equal to 0.80, 0.04, 0.08 and 0.08 (*non-contrast MRI score* classification for reader 1), based on a significance level of 0.05. Data were analyzed using STATA 16.0 software (StataCorp. 2019: StataCorp LLC) and a *p* value < 0.05 was considered statistically significant.

## Results

### Study population and adnexal mass characteristics

Overall, 1158 patients underwent gynecological MRI between 2008 and 2018 for characterization or follow-up of an adnexal lesion in our institution. A total of 291 patients were included in the study after exclusions shown in Fig. 1. Patient and lesion characteristics are listed in Table 2.

**Table 2:** Study population characteristics (whole cohort = 291 patients)

Characteristic	*N* (%)*
Age (mean ± SD)	46.8 (± 15.4)
Menopause status	
Premenopausal Postmenopausal	205 (70.4) 86 (29.6)
Pregnancy at MRI exam	10 (3.4)
CA125 value (U/mL) (median, IQR) (*n* = 254)	20 (10, 46)
Days (mean) between date of CA125 and MRI exam (median, IQR) (*n* = 254)	19.5 (10, 39)
MRI findings	
Mass size (mm) (median, IQR)	62 (42, 94)
Mass laterality	
Unilateral	242 (83.2)
Bilateral	49 (16.8)
Multiplicity for each ovary	
Single	281 (96.6)
Multiple	10 (3.4)
Total mass number per patient	
1	234 (80.4)
2	55 (18.9)
3	2 (0.7)
Ascites	27 (9.3)
Peritoneal implants	9 (3.1)
Lymphadenopathy	9 (3.1)
Pelvic only	6 (2.0)
Para-aortic	2 (0.7)
Inguinal	1 (0.3)
Management	
Surgery	250 (85.9)
Type of surgery^§^	
Ovarian cystectomy	79 (31.6)
USO only	47 (18.8)
BSO only	56 (22.4)
TAH+BSO	20 (8.0)
TAH+BSO + omentectomy	21 (8.4)
Peritoneal or ovarian biopsy	6 (2.4)
Debulking surgery	12 (4.8)
Diagnostic laparoscopy	5 (2.0)
Other^#^	4 (1.6)
Imaging follow-up (≥ 1 year)	41 (14)
Modality	
Follow-up with only US	19 (46.3)
Follow-up with only MRI	11 (26.8)
Follow-up with US+MRI	11 (26.8)
Period (median, IQR)	21 (15, 28)

*BSO* bilateral salpingo-oophorectomy, *CA125* cancer antigen 125, *IQR* interquartile range, *MRI* magnetic resonance imaging, *TAH* total abdominal hysterectomy, *US* ultrasound, *USO* unilateral salpingo-oophorectomy

*Unless otherwise specified, data are numbers of patients, with percentages in parenthesis

§The percentage of each type of surgery is calculated on the total number of surgeries (*n* = 250)

^#^Other includes cyst drainage, myomectomy or salpingectomy without other procedures

In total, 250/291 (85.9%) patients underwent surgery; the remainder were followed up with US and/or MRI over a median period of 21 months (interquartile range 15–28 months) (Table 2).

There were 297/350 (84.8%) benign and 53/350 (15.1%) malignant lesions in the whole cohort. The random subset contained 101/121 (83.4%) benign and 20/121 (16.5%) malignant lesions. Diagnoses were established using histopathology in 296/350 (84.5%) lesions in the whole cohort and 99/121 (81.8%) in the random subset, whilst imaging follow-up was used in 54/350 (15.4%) and 22/121 (18.1%) masses, respectively. The final diagnoses according to the reference standard in each cohort are given in Table 3.

**Table 3:** Final diagnosis of the adnexal masses according to the reference standard for the whole cohort and the random subset

Final diagnosis	Whole cohort (*n* = 350)	Random subset (*n* = 121)
Normal ovary, *n* (%)	6 (1.7)*	2 (1.6)
Benign disease, *n* (%)	291 (83.1)	99 (81.8)
Ovarian		
Benign Brenner tumor	2 (0.5)	0 (0.0)
Benign germ cell tumor	84 (24.0)	32 (26.4)
Benign ovarian cyst	28 (8.0)	14 (11.5)
Benign stromal tumor	29 (8.2)	8 (6.6)
Cystadenoma	53 (15.1)	14 (11.5)
Cystadenofibroma	16 (4.5)	5 (4.1)
Endometrioma	68 (19.4)	22 (18.1)
Functional cyst	3 (0.8)	1 (0.8)
TOA/inflammation	4 (1.1)	3 (2.4)
Non-ovarian		
Benign non-ovarian cyst^#^	2 (0.5)	0 (0.0)
Leiomyoma	2 (0.5)	0 (0.0)
Borderline disease, *n* (%)	14 (4.0)	5 (4.1)
Ovarian		
Serous borderline	9 (2.5)	3 (2.4)
Mucinous borderline	5 (1.4)	2 (1.6)
Malignant disease, *n* (%)	39 (11.1)	15 (12.3)
Ovarian		
Clear cell carcinoma	6 (1.7)	2 (1.6)
Endometrioid carcinoma	5 (1.4)	2 (1.6)
Serous carcinoma	16 (4.5)	6 (4.9)
Mucinous carcinoma	1 (0.2)	0 (0.0)
Transitional cell carcinoma	2 (0.5)	0 (0.0)
Malignant germ cell tumor	3 (0.8)	3 (2.4)
Malignant sex-cord stromal tumor	2 (0.5)	0 (0.0)
Metastasis^§^	2 (0.5)	0 (0.0)
Non-ovarian		
Other (i.e. lymphoma)	2 (0.5)	2 (1.6)

*TOA* tubo-ovarian abscess

*Unless otherwise specified, data are numbers of masses, with percentages in parenthesis

^#^Benign non-ovarian cyst refers to paraovarian cyst or peritoneal inclusion cyst

^§^Two adnexal masses which were scored as primary adnexal masses resulted as metastases after histopathological assessment

Amongst the benign masses (*n* = 297), 243 (81.8%) were confirmed surgically whilst 54 (18.2%) were defined as benign on the basis of stability (*n* = 26 masses) or resolution (total resolution in 15, decrease in size in 13 masses) on imaging follow-up.

The median (IQR) CA125 tumor marker level was 18 (28) and 52 (312) U/mL, in the benign (*n* = 256) and malignant (*n* = 50) groups, respectively. During surgery, 14/350 (4.0%) adnexal masses were found to be complicated with torsion. No torsion was observed in the malignant group.

### Characterization of adnexal masses with *non-contrast MRI score*

Sixty-four patients did not have an adnexal mass on MRI exam (score 1). Amongst 281 adnexal masses assigned score 2, 276 (98.2%) were benign, 2 were borderline tumors (1 borderline mucinous tumor and 1 borderline serous cystadenofibroma), 1 was a cystadenofibroma with serous carcinoma foci and 2 were clear cell carcinomas (PLR 0.1).

Amongst 12 adnexal masses assigned score 3, 9 (75%) were benign whilst 3 (25%) were borderline mucinous tumors (PLR 1.9).

**Fig. 3:**
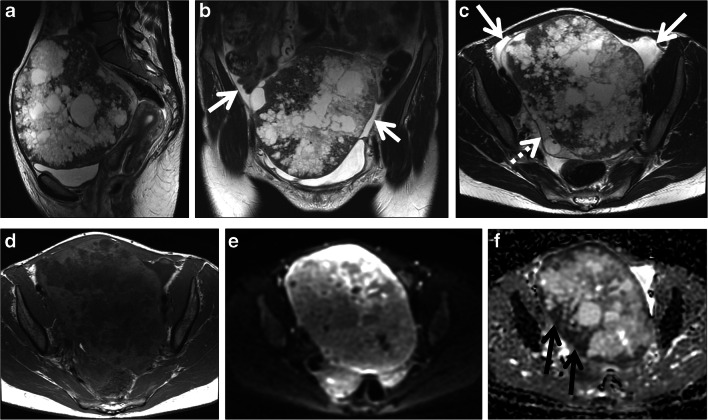
A 37-year-old woman with a pelvic mass. CA125 was 34 kU/L. Sagittal (**a**), coronal (**b**), axial T_2_-weighted (**c**) and axial T_1_-weighted (**d**) images show a large pelvic mass with solid and cystic areas. Small-volume ascites is seen around the mass (white arrows in **b** and **c**). A small amount of normal ovarian parenchyma is seen near the mass (dashed arrow in **c**). The diffusion-weighted image (*b* 800 s/mm^2^) (**e**) and ADC map (**f**) show restricted diffusion in the mass with low signal intensity areas on the ADC map (black arrows). This case was correctly classified as malignant with a score of 5 due to intermediate T_2_ signal intensity solid tissue with restricted diffusion and ascites. Histopathology showed a serous borderline ovarian tumor

Amongst 28 adnexal masses assigned score 4, 20 (71%) were malignant and 8 (29%) were benign (PLR 14). Eight of 20 malignant tumors (40%) were borderline tumors (Fig. 3). Three benign stromal tumors (ovarian leiomyoma, cellular ovarian fibroma and fibrothecoma), 3 benign germ cell tumors (struma ovarii, mature teratoma and mature teratoma with abundant thyroid tissue), 1 cystadenofibroma (endometrioid type) and 1 endometrioma with mural nodules on imaging were misclassified as malignant.

Amongst 29 adnexal masses assigned score 5, 25 (86%) were malignant and 4 (14%) were benign (ovarian fibroma, one with cellular type) (PLR 35).

**Fig. 4:**
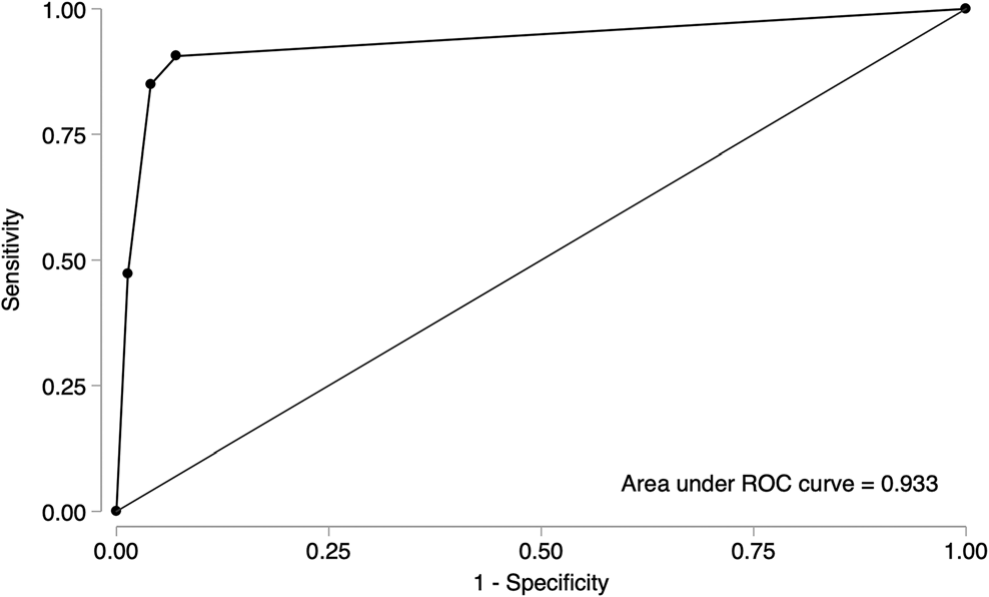
Receiver operating curve analysis of the *non-contrast MRI score* for prediction of malignancy in adnexal masses. The area under the curve (AUC) is 0.933 (95% CI 0.903–0.957)

ROC analysis of *non-contrast MRI score* for prediction of malignancy in adnexal masses revealed that the pre-defined cut-off score ≥ 4 is associated with malignancy: sensitivity 84.9% (95% CI 72.4–93.3), specificity 95.9% (95% CI 93–97.9), accuracy 94.2% (95% CI 91–96) and PLR 21 (95% CI 11.9–37). The AUC was 0.93 (95% CI 0.90–0.96) (Fig. 4).

### Reproducibility and repeatability

**Fig. 5:**
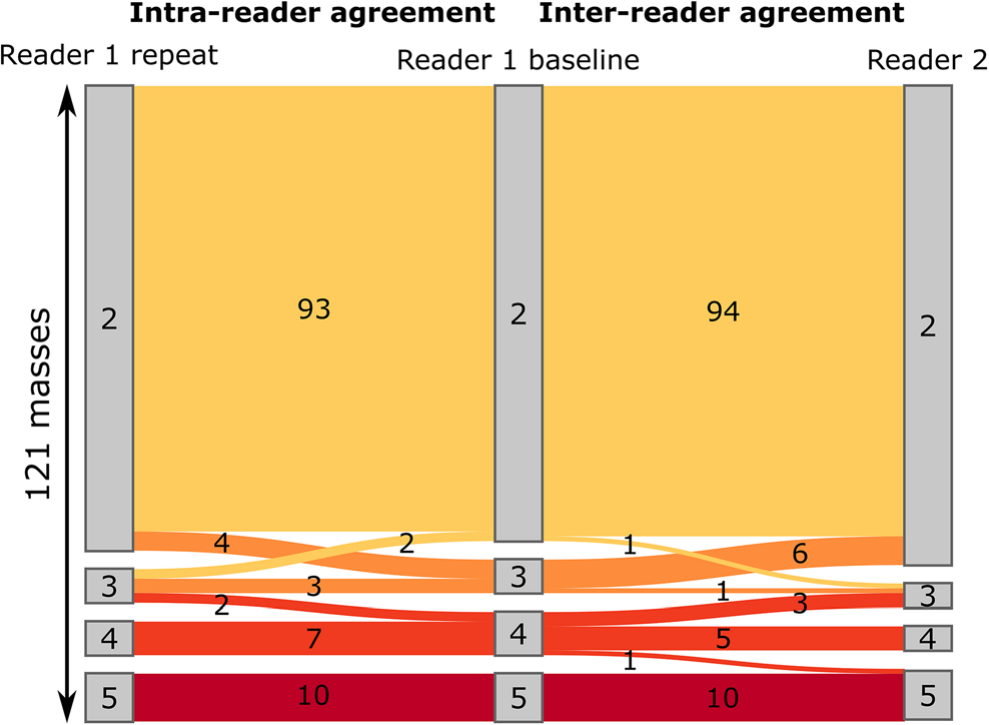
A Sankey diagram depicting the intra- and inter-reader agreement in the assessment of the *non-contrast MRI score*

There was a good inter-reader agreement for the *non-contrast MRI score* (*к* = 0.73, 95% CI 0.58–0.86). The intra-reader agreement was excellent (*к* = 0.81, 95% CI 0.70–0.88). When the weighted kappa analysis was performed to measure the importance of disagreements, both inter-reader and intra-reader agreements were excellent (*к* = 0.87, 95% CI 0.77–0.93 and *к* = 0.91, 95% CI 0.83–0.97; respectively). There was perfect agreement for masses assigned score 5 (*n* = 10). Amongst 95 masses assigned score 2 by reader1, there was disagreement in only 1 case given score 3 by reader 2, which was a borderline serous cystadenofibroma. Overall, 11/121 masses were assigned differently amongst the readers (7 benign, 2 borderline, 2 malignant) (Fig. 5, Supplementary Fig S1).

### Assessment of individual MRI features

For score 2 (benign/likely benign) lesions, 184/281 (65.4%) lesions were comprised of purely endometriotic, fatty or simple cystic masses. The distribution of individual MRI features amongst lesions is given in Table 4.

**Table 4:** Distribution of individual MRI features amongst the given *non-contrast MRI score* (*n*=350 lesions) according to assessment of reader 1

Variable	Score 2^ (*n* = 281)	Score 3 (*n* = 12)	Score 4 (*n* = 28)	Score 5 (*n* = 29)
Lesion size (mm), median (IQR)	60 (41, 86)	80 (40, 121)	66 (46, 100)	94 (53, 130)
Purely endometriotic mass	71 (25.3%)*	1 (8.3%)	0 (0.0%)	0 (0.0%)
Fatty mass	79 (28.1%)	0 (0.0%)	1 (3.6%)	0 (0.0%)
Solid mass	24 (8.5%)	4 (33.3%)	5 (17.9%)	13 (44.8%)
Simple cystic mass	34 (13.2%)	0 (0.0%)	0 (0.0%)	0 (0.0%)
Multiple septa	89 (34.6%)	5 (62.5%)	12 (52.2%)	14 (87.5%)
Thick or irregular septa^#^	-	4 (50%)	8 (36%)	12 (75%)
Cystic fluid composition				
Serous Mucinous Blood Fat Pus	77 (30.0%) 23 (8.9%) 74 (28.8%) 79 (30.7%) 4 (1.6%)	2 (25.0%) 5 (62.5%) 1 (12.5%) 0 (0.0%) 0 (0.0%)	13 (56.5 %) 1 (4.3%) 7 (30.4%) 1 (4.3%) 1 (4.3%)	12 (75.0%) 3 (18.8%) 1 (6.2%) 0 (0.0%) 0 (0.0%)
Solid tissue in the mass	55 (19.6%)	10 (83.3%)	28 (100%)	29 (100%)
Intermediate T_2_ signal of solid tissue	12 (22%)	5 (50%)	27 (96%)	29 (100%)
High DWI signal of solid tissue^a^	24 (44%)	3 (30%)	28 (100%)	29 (100%)
Diffusion restriction in the solid tissue	23 (42%)	3 (30%)	28 (100%)	29 (100%)
Ascites	8 (2.8%)	1 (8.3%)	2 (7.1%)	21 (72.4%)
Peritoneal implants	0 (0.0%)	0 (0.0%)	0 (0.0%)	13 (44.8%)
Lymphadenopathy Pelvic LAD Para-aortic LAD Inguinal LAD	2 (0.7%) 2 (0.7%) 0 (0.0%) 0 (0.0%)	0 (0.0%) 0 (0.0%) 0 (0.0%) 0 (0.0%)	0 (0.0%) 0 (0.0%) 0 (0.0%) 0 (0.0%)	8 (27.6%) 7 (24.1%) 2 (7.1%) 1 (3.4%)

*DWI* diffusion-weighted imaging, *LAD* lymphadenopathy

^In definition of *non-contrast MRI score*, scores 2, 3, 4 and 5 refer to benign/likely benign, indeterminate, suspicious and highly suspicious masses, respectively

*Unless otherwise specified, data are numbers of lesions with the relevant MRI feature, with percentages in parenthesis

^#^Thick or irregular septa was not evaluated for score 2 lesions

^a^DWI signal of the solid tissue is evaluated on high *b* value (800–1000s/mm^2^) DWI images

The MRI features of the adnexal masses all significantly discriminated benign vs. malignant (Supplementary Table S2). All the masses regarded as simple cystic mass were benign (*p* = 0.016). None of the masses including fat was malignant (*p* = 0.005). Amongst purely endometriotic masses, only 2/72 (2.7%) were malignant (*p* = 0.001). Thick or irregular septa were the least discriminating MRI features (*p* = 0.044). There was good intra-reader agreement (*к* = 0.73–1.00) and excellent inter-reader agreement (*к* = 0.85–1.00) for the individual MRI features with a perfect agreement for fatty mass, solid mass and peritoneal implants and lowest agreement for thick or irregular septa (*к* = 0.85).

### Failure analysis

The failure analysis of the cases which were incorrectly classified on imaging by either reader revealed that false-negative diagnoses occurred exclusively in lesions with mucinous and serous cystic fluid and multiple septations. False-positive diagnoses occurred primarily in purely solid masses. The association of highly discriminatory imaging features with the clinical parameters and the reference standard diagnosis of malignancy is illustrated in Supplementary Fig S2.

### Comparison between final diagnosis and specific reader diagnosis

For the whole cohort and random subset, agreement between the final diagnosis and specific diagnosis of the readers was excellent (91.4–95.9%) (Table 5). For the whole cohort, 320/350 (91.4%) masses were given the correct specific diagnosis. The readers were not able to give a specific diagnosis in 2 lesions (1 follicular lymphoma, 1 benign germ cell tumor without fat component). The inter-reader and intra-reader agreement for the specific diagnosis of the adnexal mass was excellent at 96.7% (*к* = 0.88, 95% CI 0.76–0.97). Only 4/121 masses were given different diagnoses in the random subset (2 borderline tumors, 1 benign stromal tumor and 1 benign germ cell tumor).

**Table 5:** Comparison of inter-reader and intra-reader agreements for specific diagnosis of the readers and agreement between the final diagnosis and specific reader diagnosis

Compared variables	Agreement (%)	Kappa	95% CI
Final diagnosis – reader 1* (*n* = 350)	91.4	0.70	0.62–0.79
Final diagnosis – reader 2 (*n* = 121)	95.9	0.86	0.73–0.95
Final diagnosis – reader 1_second_ (*n* = 121)	93.4	0.77	0.61–0.92
§Reader 1_first_ – reader 2 (*n* = 121)	96.7	0.88	0.77–0.97
#Reader 1_first_ – reader 1_second_ (*n* = 121)	96.7	0.88	0.76–0.97

*CI* confidence intervals

*Reader 1 has assessed the random subset at two different time points which are written as Reader1_first_ and reader 1_second_

^§^Reader 1_first_–reader 2 comparison refers to inter-reader agreement

^#^Reader 1_first_–reader 1_second_ comparison refers to intra-reader agreement

## Discussion

This study evaluated the accuracy of characterizing adnexal masses using a non-contrast MRI protocol. Benign adnexal masses constituted most cases (84.4%) which reflect our referral population for MRI characterization. In this group, our results indicate that this protocol can correctly classify adnexal masses into benign or malignant with high accuracy (94.2%) and high PLR (21) for malignancy. Additionally, there was excellent inter- and intra-reader agreement with high reproducibility and repeatability of the score. In our study, amongst adnexal masses with a score of 2, 80.7% underwent surgery, of which 97.8% were confirmed to be benign. All patients with score 3, 4 and 5 underwent surgery, with the corresponding malignancy rates as 25%, 71% and 86%, respectively. These results may therefore help to provide appropriate management stratification using morphological imaging assessment of the adnexal mass by experienced radiologists in a tertiary center.

Continuous efforts have aimed to standardize pre-operative assessment of adnexal masses in women for the last 20 years [18, 19]. Although several attempts at integrating clinical criteria, biochemical parameters and model-based US evaluation for the stratification of ovarian lesions according to malignancy have been made [20–22], only one standardized MRI scoring system for adnexal masses has been developed so far [10, 23]. This scoring system, the ADNEX MR score [10], has high sensitivity (93.5%) and specificity (96.6%), which is supported by external validation studies [13, 24, 25]. The subsequently developed O–RADS MRI score with multicenter prospective data also has excellent sensitivity, specificity, accuracy and PLR for malignancy (93%, 91%, 92% and 10.9 for experienced readers, respectively) using the same MRI protocol (except temporal resolution, 2.4 s vs. 15 s) and technique in image and data interpretation [11].

The O–RADS study therefore provides the benchmark for adnexal mass characterization [11]. The O–RADS scoring system relies upon the addition of intravenous contrast medium to assess the enhancement of the lesion using dynamic curve analysis. We recognize that there may be circumstances in which the addition of intravenous contrast medium is not possible and therefore we set out to address if an adnexal mass could be accurately characterized in a protocol without the addition of intravenous contrast medium. We have proposed a simple 5-point scoring system. This *non-contrast MRI score* is aimed to be a practical qualitative score using morphological assessment and basic comparison of tumoral signal intensities on T2, DWI and ADC map with reference to standard tissues. A proper definition of solid tissue is the principal of this score to suspect malignancy which could be clinically relevant. We achieved 84.9% sensitivity, 95.9% specificity, 94% accuracy and PLR of 21 without performing DCE-MRI. Despite the assumption that if the ADNEX MR score is utilized without dynamic contrast-enhancement, the specificity for malignancy would fall below 90% [3], we found a high specificity with our MRI protocol and image interpretation based on morphology and qualitative DWI interpretation. This also emphasizes the need for high-quality DWI in this setting. Moreover, according to PLR of malignancy, score 3 (indeterminate) in the *non-contrast MRI score* correlated to between the low- and intermediate-risk categories in O–RADS, whilst scores 4 (suspicious) and 5 (highly suspicious) corresponded to the O–RADS high-risk category. This result demonstrates that *non-contrast MRI score* may be able to interrogate further the suspicion of malignancy which may help management stratification.

There were five missed malignancies in the score 2 group in our study. These included two borderline lesions (one mucinous cystadenoma, one borderline serous cystadenofibroma), one case with microscopic serous carcinoma foci within a serous cystadenofibroma and two cases of clear cell foci arising within endometriomas. These cases demonstrate the difficulties of imaging interpretation of small foci of malignancy arising within benign lesions and are a reflection of known imaging interpretation pitfalls in complex cases such as endometriosis. It is not possible for us to speculate whether the addition of intravenous contrast medium would have allowed visualization of these foci of malignancy. But it remains an important message that imaging interpretation in borderline malignancy and endometriosis can be challenging and extra-caution is required. Although we did not aim to make a differentiation between borderline and malignant tumors, a more detailed analysis of the signal could potentially show differences between those tumors which could also be scientifically relevant as well as of translational clinical benefit. In the current literature, there are few studies that address this topic and which are focused mostly on DWI parameters [26–29].

A non-contrast protocol, with a shorter acquisition time, reducing workload for radiology departments, is highly desirable in high volume referral hospitals and in patients where a shorter protocol is likely to confer a diagnostic study. Our proposed non-contrast MRI protocol (average acquisition times 18 min and 12 min for 1.5 T and 3.0 T, respectively) may enable diagnostic centers to save time, which may be critical in some instances, especially at high magnetic field strengths. Furthermore, the use of GBCAs is not without risk and it increases the total cost of the study [30]. These issues also make a non-contrast MRI protocol preferable. Concordantly, our results support that in instances when the administration of intravenous contrast medium is not possible, not preferred or should be avoided, a non-contrast MRI protocol and scoring system can safely classify adnexal masses into benign or malignant with high reproducibility and repeatability.

Our study reached excellent inter- and intra-reader agreement for the specific diagnosis of adnexal masses. The study of Thomassin-Naggara et al showed that individual MRI features of adnexal masses show variable inter-reader agreement which was lowest in the assessment of grouped and thickened septa [10]. Similarly, our study found the lowest inter- and intra-reader agreement for thick or irregular septa, reflecting the difficulties in septa evaluation. Our results also demonstrated that septa evaluation: namely multiple septa and thick irregular septa gave a lower intra-reader agreement than inter-reader agreement also reflecting that this is a difficult area of evaluation. Although kappa values for intra-reader agreement were over 0.70 for those features, our results show that evaluation of these features may sometimes be challenging. We believe that this is an important limitation to recognize as these features can be difficult to use as markers of malignancy as benign lesions may have multiple septae but due to the overall morphology and signal characteristics, the radiologist can interpret the diagnosis correctly as a cystadenofibroma for example. Discrepancies gain importance when individual MRI features are used in structured reporting or as decision criteria of a scoring system. In our opinion, looking at overall morphology to make a diagnosis is more valuable; this was the case in our study where the agreement rates were over 95%. When the readers assessed the case as a whole and gave a specific diagnosis, the inter-reader and intra-reader agreements were very high (kappa 0.88). We believe that this result supports the radiologist’s evaluation of the entirety of the lesion as a whole rather than too much reliance on single factors.

Our study has several limitations. Firstly, it was a retrospective single-center study. Secondly, long-term follow-up (i.e. more than 2 years) was not available for patients who did not undergo surgery. However, median imaging follow-up was 21 months and lesions were considered benign if they resolved, decreased in size or stayed stable. Thirdly, the distribution of types of masses differed slightly from previous scoring studies [10, 11]. Although we had fewer malignant lesions in comparison to the ADNEX [10] and O–RADS [11] studies (15.1% vs. 18.8% vs. 18.4%, respectively), the percentage of borderline cases, which can create challenges, was pretty similar (4% vs. 3.6% vs. 3%, respectively). Still, our proposed score achieved high accuracy (94% vs. 96% vs. 92%, respectively). Fourth, the assessment was done by readers with experience in gynecologic oncologic imaging which may create a limitation in global standardization of this score. Nevertheless, our results suggest that given the appropriate training and support, qualitative radiology reporting and assessment of adnexal masses could potentially be taught to radiologists reporting these studies. Lastly, an external validation of our proposed non-contrast scoring was not performed, and such validation would be crucial to support future adoption of this score into wider clinical practice.

In conclusion, our study shows that non-contrast MRI has high accuracy and excellent inter- and intra-reader agreement for characterization of adnexal masses. This suggests that a morphological and qualitative DWI assessment by radiologists with experience in gynecological imaging can be an alternative to safely guide patient management when intravenous contrast medium and a dynamic curve assessment for the formal O–RADS score cannot be provided.

## Supplementary information

ESM 1 (DOCX 1461 kb)

## Abbreviations

ADC: Apparent diffusion coefficient
CA125: Cancer antigen 125
DCE: Dynamic contrast-enhanced
DWI: Diffusion-weighted imaging
GBCA: Gadolinium-based contrast agent
MR(I): Magnetic resonance (imaging)
O–RADS: Ovarian–Adnexal Imaging–Reporting and Data System
SI: Signal intensity
US: Ultrasound

## References

[1] Timmerman D, Schwarzler P, Collins WP (1999). Subjective assessment of adnexal masses with the use of ultrasonography: an analysis of interobserver variability and experience. Ultrasound Obstet Gynecol.

[2] Forstner R, Meissnitzer M, Cunha TM (2016). Update on imaging of ovarian cancer. Curr Radiol Rep.

[3] Sadowski EA, Rockall AG, Maturen KE, Robbins JB, Thomassin-Naggara I (2019). Adnexal lesions: imaging strategies for ultrasound and MR imaging. Diagn Interv Imaging.

[4] Atri M, Alabousi A, Reinhold C, Expert Panel on Women’s Imaging (2019). ACR Appropriateness Criteria® Clinically suspected adnexal mass, no acute symptoms. J Am Coll Radiol.

[5] Anthoulakis C, Nikoloudis N (2014). Pelvic MRI as the “gold standard” in the subsequent evaluation of ultrasound-indeterminate adnexal lesions: a systematic review. Gynecol Oncol.

[6] Forstner R, Thomassin-Naggara I, Cunha TM (2017). ESUR recommendations for MR imaging of the sonographically indeterminate adnexal mass: an update. Eur Radiol.

[7] Bazot M, Daraï E, Nassar-Slaba J, Lafont C, Thomassin-Naggara I (2008). Value of magnetic resonance imaging for the diagnosis of ovarian tumors: a review. J Comput Assist Tomogr.

[8] Medeiros LR, Freitas LB, Rosa DD (2011). Accuracy of magnetic resonance imaging in ovarian tumor: a systematic quantitative review. Am J Obstet Gynecol.

[9] Allen BC, Hosseinzadeh K, Qasem SA, Varner A, Leyendecker JR (2014). Practical approach to MRI of female pelvic masses. AJR Am J Roentgenol.

[10] Thomassin-Naggara I, Aubert E, Rockall A (2013). Adnexal masses: development and preliminary validation of an MR imaging scoring system. Radiology.

[11] Thomassin-Naggara I, Poncelet E, Jalaguier-Coudray A (2020). Ovarian-adnexal reporting data system magnetic resonance imaging (O-RADS MRI) score for risk stratification of sonographically indeterminate adnexal masses. JAMA Netw Open.

[12] American College of Radiology (ACR) (2020) Ovarian-Adnexal Reporting and Data System (O–RADS). American College of Radiology (ACR) Web site. Available via https://www.acr.org/Clinical-Resources/Reporting-and-Data-Systems/O-Rads#MRI. Accessed 6 June 2020

[13] Pereira PN, Sarian LO, Yoshida A (2018). Accuracy of the ADNEX MR scoring system based on a simplified MRI protocol for the assessment of adnexal masses. Diagn Interv Radiol.

[14] Pereira PN, Sarian LO, Yoshida A et al (2019) Improving the performance of IOTA simple rules: sonographic assessment of adnexal masses with resource-effective use of a magnetic resonance scoring (ADNEX MR scoring system). Abdom Radiol (NY). 10.1007/s00261-019-02207-9 10.1007/s00261-019-02207-9 31482379

[15] Ramalho J, Ramalho M, Jay M, Burke LM, Semelka RC (2016). Gadolinium toxicity and treatment. Magn Reson Imaging.

[16] The Royal College of Radiologists (2018) RCR position statement on the revision of marketing authorisations for gadolinium based contrast agents. Available via https://www.rcr.ac.uk/posts/rcr-position-statement-revision-marketing-authorisations-gadolinium-based-contrast-agents. Accessed 26 March 2020

[17] The Royal College of Radiologists (2019). Guidance on gadolinium-based contrast agent administration to adult patients. Available via https://www.rcr.ac.uk/publication/guidance-gadolinium-based-contrast-agent-administration-adult-patients. Accessed 26 March 2020

[18] Andreotti RF, Timmerman D, Benacerraf BR (2018). Ovarian-Adnexal reporting lexicon for ultrasound: a white paper of the ACR Ovarian-Adnexal Reporting and Data System Committee. J Am Coll Radiol.

[19] Tong A (2020). Differentiating benign and malignant adnexal masses: work still in progress. Diagn Interv Imaging.

[20] Timmerman D, Testa AC, Bourne T (2005). Logistic regression model to distinguish between the benign and malignant adnexal mass before surgery: a multicenter study by the International Ovarian Tumor Analysis Group. J Clin Oncol.

[21] Rossi A, Braghin C, Soldano F (2011). A proposal for a new scoring system to evaluate pelvic masses: Pelvic Masses Score (PMS). Eur J Obstet Gynecol Reprod Biol.

[22] Meys EM, Kaijser J, Kruitwagen RF (2016). Subjective assessment versus ultrasound models to diagnose ovarian cancer: a systematic review and meta-analysis. Eur J Cancer.

[23] Sadowski EA, Robbins JB, Rockall AG, Thomassin-Naggara I (2018). A systematic approach to adnexal masses discovered on ultrasound: the ADNEx MR scoring system. Abdom Radiol (NY).

[24] Ruiz M, Labauge P, Louboutin A, Limot O, Fauconnier A, Huchon C (2016). External validation of the MR imaging scoring system for the management of adnexal masses. Eur J Obstet Gynecol Reprod Biol.

[25] Sasaguri K, Yamaguchi K, Nakazono T (2019). External validation of ADNEX MR SCORING system: a single-centre retrospective study. Clin Radiol.

[26] Miramura R, Kato F, Tha KK (2016). Comparison between borderline ovarian tumors and carcinomas using semi-automated histogram analysis of diffusion-weighted imaging: focusing on solid components. Jpn J Radiol.

[27] Lu JJ, Pi S, Ma FH, Zhang GF, Wei Qiang J (2019). Apparent diffusion coefficients measured using different regions of interest in differentiating borderline from malignant ovarian tumors. Acta Radiol.

[28] Lu J, Pi S, Ma FH (2019). Value of normalized apparent diffusion coefficients in differentiating between borderline and malignant epithelial ovarian tumors. Eur J Radiol.

[29] He M, Song Y, Li H (2020). Histogram analysis comparison of monoexponential advanced diffusion-weighted imaging, and dynamic contrast-enhanced MRI for differentiating borderline from malignant epithelial ovarian tumors. J Magn Reson Imaging.

[30] Schieda N, Krishna S, Davenport MS (2019). Update on Gadolinium-based contrast agent-enhanced imaging in the genitourinary system. AJR Am J Roentgenol.

