# Supplementary material for: Non-contrast MRI can accurately characterize adnexal masses: a retrospective study
Source: Eur Radiol. 2021 Mar 16;31(9):6962–73. doi: 10.1007/s00330-021-07737-9 (PMC8379126; doi:10.1007/s00330-021-07737-9)

**Supplementary Table 1** Non-contrast MRI protocol for adnexal mass characterization and scanning parameters

|  |  |  | **1.5T** |  |  |  |
| --- | --- | --- | --- | --- | --- | --- |
| **Parameter** | **Sagittal T_2_WI** | **Axial**  **T_2_WI** | **Coronal T_2_WI** | **Axial**  **T_1_WI** | **Axial**  **T_1_WI +FS*** | **Axial**  **DWI** |
| TR (ms) | 4144 | 3464 | 4323 | 653 | 6.2 | 7228 |
| TE (ms) | 80 | 80 | 83 | Min full (8.8) | Min Full (8.8) | Minimum (69.5) |
| Echo train length | 23 | 23 | 23 | 4 | - | 1 |
| Flip angle (◦) | 160 | 160 | 160 | 160 | 12 | - |
| Number of averages | 2 | 2 | 2 | 2 | 2 | 10 |
| FOV (cm) | 26 | 24 | 28 | 24 | 32 | 38 |
| Matrix size | 320x224 | 352x224 | 352x224 | 320x224 | 288x224 | 128x128 |
| Slice thickness (mm) | 4 | 4 | 4 | 4 | 5 | 4.5 |
| Slice interval (mm) | 0.4 | 0.4 | 0.4 | 0.4 | 0 | 0 |
| *b* value (s/mm^2^) | - | - | - | - | - | 0, 800-1000 |
| Acquisition time (min:s) | 2:54 | 3:39 | 3:02 | 4:06 | 0:30 | 3:51 |
| **3T** | | | | | | |
| **Parameter** | **Sagittal T_2_WI** | **Axial**  **T_2_WI** | **Coronal T_2_WI** | **Axial**  **T_1_WI** | **Axial**  **T_1_WI +FS*** | **Axial**  **DWI** |
| TR (ms) | 2500 | 2500 | 3208 | 400 | 4.5 | 3733.0 |
| TE (ms) | 102 | 102 | 102 | Min Full (9.1) | Min Full  (1.4) | Minimum  (64.8) |
| Echo train length | 16 | 16 | 14 | 4 | - | - |
| Flip angle (◦) | 111 | 111 | 111 | 111 | 12 | - |
| Number of averages | 3 | 3 | 3 | 2 | 2 | 10 |
| FOV (cm) | 24 | 24 | 24 | 24 | 32 | 28 |
| Matrix size | 384x288 | 384x288 | 384x288 | 384x256 | 296x296 | 128x128 |
| Slice thickness (mm) | 4 | 4 | 4 | 4 | 4.2 | 4 |
| Slice interval (mm) | 1 | 1 | 1 | 1 | 1 | 0 |
| *b* value (s/mm^2^) | - | - | - | - | - | 0, 800-1000 |
| Acquisition time (min:s) | 2:03 | 2:18 | 3:26 | 0:54 | 0:48 | 2:03 |

DWI = diffusion-weighted imaging*,* FOV = field of view*,* FS = fat suppressed, TE = echo time*,* TR = repetition time, WI = weighted imaging

*Axial T1WI+FS images were acquired with LAVA-Flex sequence which is a 3D Dual Echo sequence that provides in-phase, opposed-phase, water contrast and fat contrast images in one scan.

**Supplementary Table 2** Comparison of MRI features and tumor characteristics (benign vs. malignant). Inter-reader and intra-reader agreement results for the random subset (*n*=121 masses) are given for each feature.

| **MRI feature** | **Benign**  **(*n*=297)** | **Malignant**  **(*n*=53)** | **Total**  **(*n*=350)** | ***p* value** | **Inter-reader agreement (*к*)** | **Intra-reader agreement (*к*)** |
| --- | --- | --- | --- | --- | --- | --- |
| Lesion size, mm * | 60 (41, 87) | 80 (51, 112) | 62 (42, 94) | 0.010 | - | - |
| Simple cystic mass^#^  No  Yes | 230 (87.1)  34 (12.9) | 40 (100)  0 (0.0) | 270 (88.8)  34 (11.2) | 0.016 | 0.96 | 0.86 |
| Purely endometriotic mass  No  Yes | 227 (76.4)  70 (23.6) | 51 (96.2)  2 (3.8) | 278 (79.4)  72 (20.6) | 0.001 | 0.95 | 1.00 |
| Fatty mass  No  Yes | 217 (73.1)  80 (26.9) | 53 (100)  0 (0) | 270 (77.1)  80 (22.9) | 0.005 | 1.00 | 1.00 |
| Solid mass  No  Yes | 264 (88.9)  33 (11.1) | 40 (75.5)  13 (24.5) | 304 (86.9)  46 (13.1) | 0.008 | 1.00 | 1.00 |
| Multiple septa  No  Yes | 172 (65.2)  92 (34.8) | 12 (30.0)  28 (70.0) | 184 (60.5)  120 (39.5) | <0.001 | 0.95 | 0.81 |
| Thick or irregular septa  No  Yes | 9 (75.0)  3 (25.0) | 13 (38.2)  21 (61.8) | 22 (47.8)  24 (52.2) | 0.044 | 0.85 | 0.73 |
| Cystic fluid composition  Serous  Mucinous  Blood  Fat  Pus | 81 (30.7)  24 (9.1)  75 (28.4)  80 (30.3)  4 (1.5) | 24 (58.5)  8 (19.5)  8 (19.5)  0 (0)  1 (2.5) | 105 (34.4)  32 (10.5)  83 (27.2)  80 (26.2)  5 (1.7) | <0.001 | 0.91 | 0.92 |
| Solid tissue  No  Yes | 225 (75.8)  72 (24.2) | 3 (5.7)  50 (94.3) | 228 (65.1)  122 (34.9) | <0.001 | 0.94 | 0.94 |
| T2 signal of solid tissue  Low  Intermediate | 45 (62.5)  27 (37.5) | 4 (8.0)  46 (92.0) | 49 (40.2)  73 (59.8) | <0.001 | 0.94 | 1.00 |
| DWI signal of solid tissue  Low  High | 35 (48.6)  37 (51.4) | 5 (10.0)  45 (90.0) | 40 (32.8)  82 (67.2) | <0.001 | 0.89 | 0.83 |
| Solid tissue diffusion restriction  No  Yes | 36 (50.0)  36 (50.0) | 6 (12.0)  44 (88.0) | 42 (34.4)  80 (65.6) | <0.001 | 1.00 | 0.88 |
| Ascites  No  Yes | 286 (96.3)  11 (3.7) | 32 (60.4)  21 (39.6) | 318 (90.8)  32 (9.1) | <0.001 | 0.96 | 0.76 |
| Peritoneal implants  No  Yes | 297 (100)  0 (0) | 40 (75.5)  13 (24.5) | 337 (96.2)  13 (3.7) | <0.001 | 1.00 | 1.00 |
| Lymphadenopathy  No  Yes | 294 (99.0)  3 (1.0) | 46 (86.8)  7 (13.2) | 340 (97.1)  10 (2.8) | <0.001 | 1.00 | 0.85 |
| Pelvic lymphadenopathy  No  Yes | 294 (99.0)  3 (1.0) | 47 (88.7)  6 (11.3) | 341 (97.4)  9 (2.6) | 0.001 | 1.00 | 0.80 |
| Para-aortic lymphadenopathy^  No  Yes | 297 (100)  0 (0) | 50 (96.2)  2 (3.8) | 347 (99.1)  2 (0.6) | 0.022 | 1.00 | 1.00 |
| Inguinal lymphadenopathy  No  Yes | 297 (100)  0 (0) | 52 (98.1)  1 (1.9) | 349 (99.7)  1 (0.3) | 0.15 | 1.00 | 1.00 |

*DWI* diffusion-weighted imaging*, MRI* magnetic resonance imaging

*Lesion size is given as median (Inter Quartile Range)

^#^Unless otherwise specified, data are numbers of masses, with percentages in parenthesis

^Para-aortic lymphadenopathy was not evaluated in one case due to severe degradation of images with motion artefacts.

**Supplementary Fig S1.** 60 year-old woman with left adnexal mass. CA125 was 4 kU/L. Sagittal (**a**), axial T_2_-weighted (**b**), axial T_1_-weighted (**c**) and fat-suppressed T_1_-weighted (**d**) images show a complex left adnexal mass. Fat signal is not seen in the mass. The diffusion-weighted image (b 800 s/mm^2^) (**e**) and ADC map (**f**) show significantly restricted diffusion in the mass with low signal intensity on the ADC map. There was discrepancy in scoring of this case as one of the readers assigned a score of 4 (suspicious for malignancy) and the other a score of 3 (indeterminate). Histopathology showed a benign mature cystic teratoma with keratin content.

a
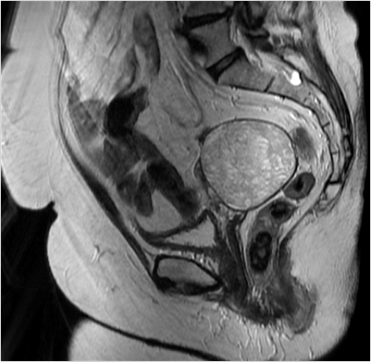
 b
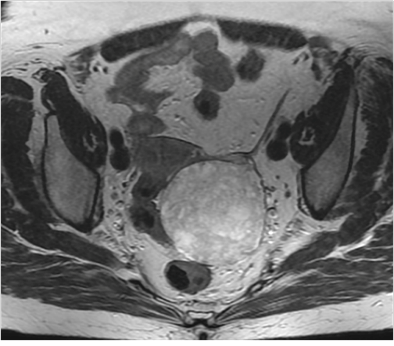
 c
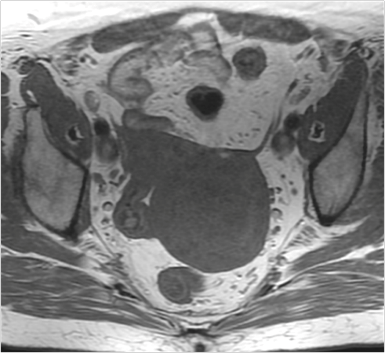


d
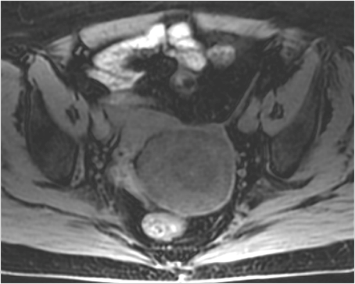
 e
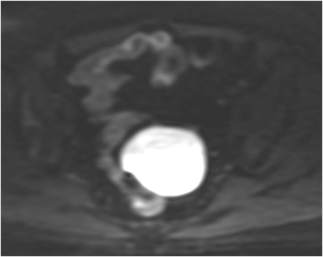
 f
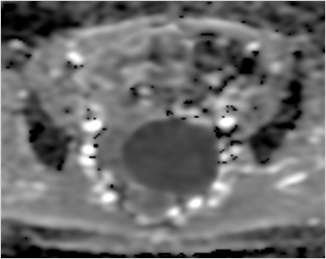


**Supplementary Figure S2.** This circos plot illustrates the distribution of patient and imaging characteristics in the random subset and highlights disagreements between the reference-standard (pathology in 99 and imaging follow-up in 22 lesions) and the two readers. The benign versus malignant categorization of the readers' assessment is based on dichotomized variables of non-contrast MRI score. Clinical parameters are plotted on a violet background and malignant cases had higher prevalence of raised CA125 levels. Imaging characteristics are plotted on a blue background and a higher prevalence of intermediate solid tissue T2 intensity and peritoneal implants can be seen in malignant lesions. Red lines indicate disagreements between the final diagnosis and each reader as well as among the two readers. False negative diagnosis on imaging occurred exclusively in patients with mucinous and serous cystic fluid and multiple septations. False positive diagnoses were observed primarily in patients with purely solid, intermediately T2 intense masses with true diffusion restriction.


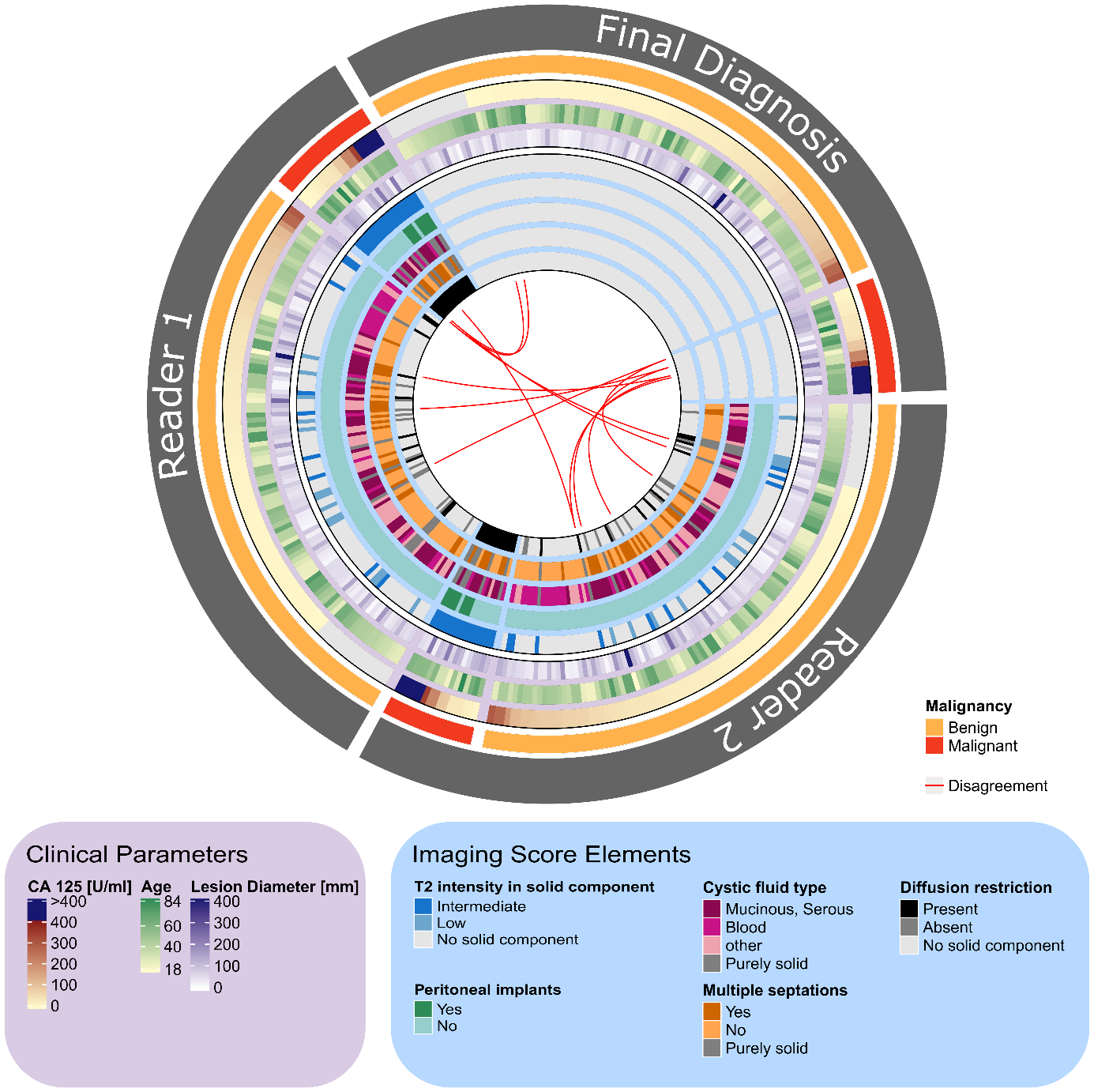

Supplement: Supplementary file 1 — (DOCX 1461 kb) [file 330_2021_7737_MOESM1_ESM.docx]
